# Multi‐analyte measurement of blood biomarkers for Alzheimer's disease: enabling concurrent analysis of affected pathophysiological processes

**DOI:** 10.1002/alz.088410

**Published:** 2025-01-09

**Authors:** Xuemei Zeng, Tara K Lafferty, Anuradha Sehrawat, Yijun Chen, Pamela C.L. Ferreira, Bruna Bellaver, Guilherme Povala, M. Ilyas Kamboh, William E Klunk, Oscar L. Lopez, Tharick A. Pascoal, Victor L Villemagne, Mary Ganguli, Ann D Cohen, Thomas K Karikari

**Affiliations:** ^1^ University of Pittsburgh, Pittsburgh, PA USA; ^2^ Department of Human Genetics, University of Pittsburgh, Pittsburgh, PA USA; ^3^ Department of Psychiatry, School of Medicine, University of Pittsburgh, Pittsburgh, PA USA

## Abstract

**Background:**

Alzheimer’s disease (AD) is a multifaceted condition associated with various brain pathologies, necessitating diverse biomarkers for precise prognosis, diagnosis, clinical management, and therapeutic development/evaluation. The integration of multiple biomarkers into a single test can enhance efficiency, reduce analytical errors, and save on specimen volume. Alamar Biosciences recently introduced the NULISAseq CNS disease panel, a multiplex NUcleic acid‐linked Immuno‐Sandwich Assay (NULISA) targeting ∼120 analytes. This includes classical amyloid, tau, and neurodegeneration biomarkers and allows for key proteins defining various disease hallmarks. However, its performance in assessing AD pathologies remains undetermined.

**Method:**

The NULISAseq CNS disease panel was applied to 264 plasma samples from two independent cohorts (Human Connectome and MYHAT‐NI), encompassing cross‐sectional and longitudinal specimens. Wilcoxon rank sum tests and Spearman rank‐based correlations were used to determine the significance of the association between NULISAseq measurements and neuroimaging‐based assessment of AD pathologies. Classical AD biomarkers (p‐tau181, p‐tau217, p‐tau231, GFAP, NEFL, and amyloid beta peptides Aβ40 and Aβ42), along with cytokines IL6 and TNF, were also measured using alternative immunoassays (Simoa and Quest) in a subset of samples for cross‐platform validation.

**Result:**

The NULISAseq CNS disease panel measured a total of 116 targets with robust reproducibility, showing mean intra‐plate and inter‐plate coefficients of variation (CVs) of 5.2% and 7.6%, respectively. Consistent concordance was observed between NULISAseq measurements and those obtained with Simoa or Quest assays, with Spearman’s correlation coefficients up to 0.874. NULISAseq measurements demonstrated strong predictive capability for amyloid burden and neurodegenerative status and, to a lesser extent, for tau pathology. Specifically, NULISAseq measurements of p‐tau217, p‐tau231, and GFAP exhibited strong correlations with amyloid burden, while GFAP and NEFL displayed robust correlations with neurodegeneration. In addition to these classical AD biomarkers, several NULISAseq targets, such as TIMP3, SOD1, and SQSTM1, demonstrated significant associations with AD pathologies.

**Conclusion:**

The NULISAseq CNS panel provides reliable and comprehensive measurement of classical AD biomarkers and other key proteins in neurodegenerative diseases, establishing it as a valuable tool for AD biomarker discovery and validation studies. Concurrent evaluation of markers will be critical to the implementation of the revised AT(N) framework to include an expanded set of multiple biological processes.

